# Insecticide resistance profiles of *Anopheles arabiensis* and relationship with *Microsporidia MB* infection in two rice agroecosystems in Kenya

**DOI:** 10.1186/s13071-025-07212-0

**Published:** 2026-01-22

**Authors:** Winfred K. Mutwiri, Ephantus J. Muturi, Josephine W. Ngunjiri, Jackson W. Muthengia, Nancy L. M. Budambula, Joshua K. Muli, Genson Murithi, Franklin N. Nyabuga, Njogu M. Kimani, Moses M. Muraya, David P. Tchouassi

**Affiliations:** 1 International Centre of Insect Physiology and Ecology, Nairobi, Kenya; 2 Chuka University, Chuka, Kenya; 3 U.S. Department of Agriculture, Agricultural Research Service, Crop Bioprotection Research Unit, National Center for Agricultural Utilization Research, Peoria, IL USA; 4 University of Embu, Embu, Kenya

**Keywords:** Malaria vectors, Insecticide resistance, Irrigation, Pyrethroids, *Microsporidia MB*

## Abstract

**Background:**

Insecticide resistance monitoring in vector populations is a key pillar of the Global Plan for Insecticide Resistance Management in malaria vectors. This study assessed the susceptibility of *Anopheles arabiensis* populations from Mwea and Ahero, Kenya to six insecticides. The association between insecticide resistance and *Microsporidia MB* infection, a symbiont known to block malaria transmission in *An. arabiensis* was also investigated.

**Methods:**

Mosquitoes were exposed to permethrin, deltamethrin, alphacypermethrin, malathion, bendiocarb, and dichlorodiphenyltrichloroethane (DDT) using the Centers for Disease Control and Prevention (CDC) bottle bioassay. Resistance intensity and synergist bioassays for pyrethroids were conducted to evaluate the strength of resistance and the contribution of cytochrome P450s to pyrethroid resistance. *Microsporidia MB* infection was detected and quantified using qPCR.

**Results:**

A total of 3120 females were tested. Populations from both study sites were susceptible to bendiocarb but resistant to all three pyrethroids. Mortality rates following exposure to alpha-cypermethrin, permethrin, and deltamethrin respectively were 0%, 4.7%, and 25.7% in Ahero, and 25.7%, 6.2%, and 26.6% in Mwea. Mortality increased with increasing permethrin concentration with 1 × , 2 × , 5 × , and 10 × values of 4.7%, 17.2%, 70.8%, and 84.4% respectively in Ahero and 6.2%, 29.4%, 85.3%, and 100% in Mwea. The Ahero population was susceptible to malathion but had reduced susceptibility to DDT (92.7%) while the Mwea population was susceptible to DDT and resistant to malathion (69.2%). Pre-exposure to piperonyl butoxide fully restored pyrethroid susceptibility in the Mwea population, indicating metabolic resistance and partially restored permethrin susceptibility (4.7 to 86.7%) in Ahero population, indicating the presence of other resistance mechanisms. *Microsporidia MB* was detected in Ahero population and mean (± se) infection density was significantly higher in mosquitoes that survived 2 × and 5 × permethrin doses (1017.6 ± 296.6) compared with those that succumbed to these doses (171.3 ± 78.0).

**Conclusions:**

*Anopheles arabiensis* populations from the two sites exhibit heterogeneous yet high levels of insecticide resistance, particularly to pyrethroids. The findings highlight the need to incorporate synergist-based interventions into resistance management strategies. This study is the first to document an association between *Microsporidia MB* density and the intensity of insecticide resistance in *An. arabiensis*, and further studies are needed to clarify this relationship and its significance to malaria control.

**Graphical abstract:**

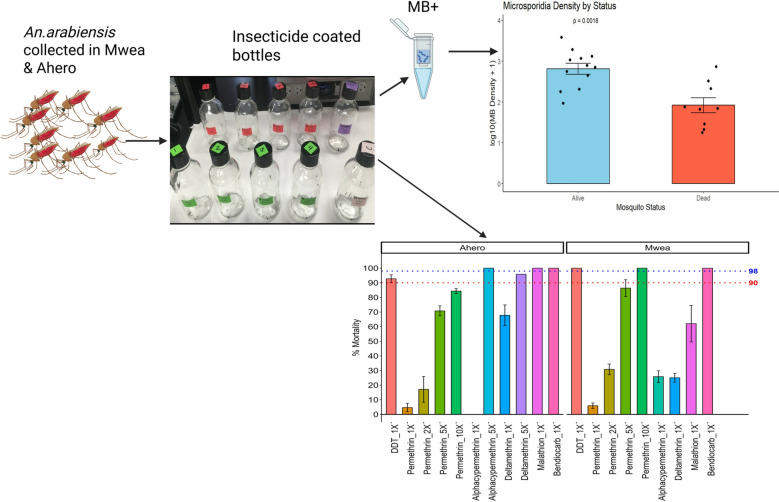

**Supplementary Information:**

The online version contains supplementary material available at 10.1186/s13071-025-07212-0.

## Background

Malaria remains one of the most important vector-borne diseases in sub-Saharan Africa, accounting for approximately 90% of the global malaria burden. Vector control is a fundamental component of malaria prevention and relies heavily on insecticide-based interventions, particularly long-lasting insecticidal nets (LLINs) and indoor residual spraying (IRS) [1, 2]. These strategies have significantly reduced malaria transmission, but their continued effectiveness in malaria control remains a concern owing to widespread resistance of malaria vectors to insecticides. Understanding the pattern of insecticide resistance in local mosquito populations is crucial for the development of evidence-based resistance management strategies. These strategies may include deployment of new generation LLINs that incorporate dual active ingredients or synergists, such as piperonyl butoxide (PBO), to enhance effectiveness against resistant mosquitoes [3, 4].

Pyrethroids are the primary insecticides used in LLINs and are also widely applied in IRS, alongside other insecticide classes such as carbamates, organophosphates, organochlorines, and more recently neonicotinoids such as clothianidin [5]. These chemicals are also extensively used in agriculture, exerting additional selection pressure on malaria vectors. Resistance to all four major insecticide classes has been reported in the main vectors of malaria in Africa including *Anopheles coluzzi, An. funestus*, *An. gambiae* s.s., and *An. arabiensis* [6]. This resistance is often more prevalent and intense in areas with high agricultural pesticide use [7, 8].

The two main mechanisms of insecticide resistance in malaria vectors are metabolic detoxification and target site insensitivity [6]. Metabolic resistance involves the elevated activity of detoxifying enzymes, including cytochrome P450 monooxygenases, esterases, and glutathione S-transferases [6]. Target-site resistance arises from point mutations in genes encoding proteins targeted by insecticides, such as voltage-gated sodium channels, which confer knockdown resistance (*kdr*) to pyrethroids and DDT. The mutation involves a leucine (TTA) to phenylalanine (TTT) transversion at codon 1014 of the voltage-gated sodium channel gene (L1014F), while the L1014S mutation results from a leucine (TTA) to serine (TCA) transition at the same codon. The L1014F mutation has been reported exclusively in West Africa, whereas L1014S occurs in both West and East Africa, including Kenya [9]. Other mutations such as the acetylcholinesterase (AChE), specifically the Ace-1 G119S substitution, render vectors insensitive to organophosphate and carbamate by altering acetylcholinesterase. Such mutations have been reported in several regions of sub-Saharan Africa [10]. Additional resistance mechanisms have also been reported, including cuticular thickening that reduces insecticide penetration, behavioral avoidance of treated surfaces [11, 12], and microbe-mediated resistance [13, 14]. However, the geographical distribution of these mechanisms remains poorly understood, thus limiting the ability to design tailored vector control strategies.

Irrigated rice cultivation presents a unique challenge for malaria control efforts. Inundated rice fields support high densities of malaria vectors, influencing malaria transmission risk, and intensifying reliance on insecticide-based vector control. The routine use of pesticides to manage rice pests and diseases can also expose the mosquito larvae to additional selection pressure that may accelerate resistance development [15, 16]. Understanding the dynamics of insecticide resistance and the underlying mechanisms in malaria vectors in these agroecosystems is essential for the design and implementation of effective resistance management interventions.

Another emerging concern is the impact of insecticide resistance on malaria transmission dynamics. While resistance alleles provide a survival advantage in the presence of insecticides, they may impose fitness costs when selection pressure is absent [17]. These fitness costs may manifest as altered immunity or vector competence, affecting the mosquito’s ability to host or transmit *Plasmodium* parasites [18, 19]. In arid and semi-arid regions of East Africa, including irrigated rice growing areas, *An. arabiensis* is the predominant malaria vector [20, 21]. Recent studies have shown that *An. arabiensis* populations can carry a vertically transmitted microsporidian endosymbiont known as *Microsporidia MB*, which can impair mosquito ability to transmit malaria parasites [22]. A growing body of knowledge suggests that microbes can mediate insecticide resistance in mosquitoes [13, 23–25]. These studies have primarily focused on insecticide resistance mediated by bacteria. However, when DDT and pyrethroid-resistant *An. gambiae* were infected with the microsporidian parasite, *Vavraia culicis,* they became more susceptible to DDT and permethrin [26]. These findings suggest that microsporidian parasites can modulate insecticide resistance, but our understanding of the link between *Microsporidia MB* and insecticide resistance in malaria vectors remains poorly understood.

The objective of this study was to assess the status of insecticide susceptibility in *An. arabiensis* populations from two irrigated rice agroecosystems, Mwea and Ahero rice irrigation schemes, in central and western Kenya, respectively. These sites represent distinct malaria epidemiological zones. Ahero is a malaria-endemic region characterized by perennial transmission and consistently high malaria incidence, while Mwea has seasonal transmission with relatively few malaria cases reported annually [27]. Earlier studies conducted in Mwea and Ahero reported no resistance to World Health Organization (WHO)-recommended insecticide classes used for IRS and LLINs, including pyrethroids [28]. However, more recent investigations have documented moderate-to-high levels of pyrethroid resistance, while susceptibility to bendiocarb, DDT, clothianidin, pirimiphos-methyl, and chlorfenapyr has been retained [27–30]. The emergence of resistance in these regions may be partly attributed to increased coverage and use of ITNs and IRS particularly during the scale-up campaigns led by the Ministry of Health between 2002 and 2004. Furthermore, intensive use of agricultural pesticides has been recognized as an additional major driver of resistance development in local mosquito populations [31]. Mosquito populations were tested for susceptibility to six insecticides spanning four chemical classes: permethrin, deltamethrin, and alphacypermethrin (pyrethroids); bendiocarb (carbamate); DDT (organochlorine); and malathion (organophosphate). Resistance intensity and synergist assays were also conducted for the three pyrethroids to determine the strength of resistance and the contribution of cytochrome P450 monooxygenases. We also evaluated the relationship between *Microsporidia MB* and permethrin resistance in the two mosquito populations. The findings of this study are crucial for the development of evidence-based insecticide resistance management strategies in irrigated rice agroecosystems.

## Methods

### Study area

**Fig. 1:**
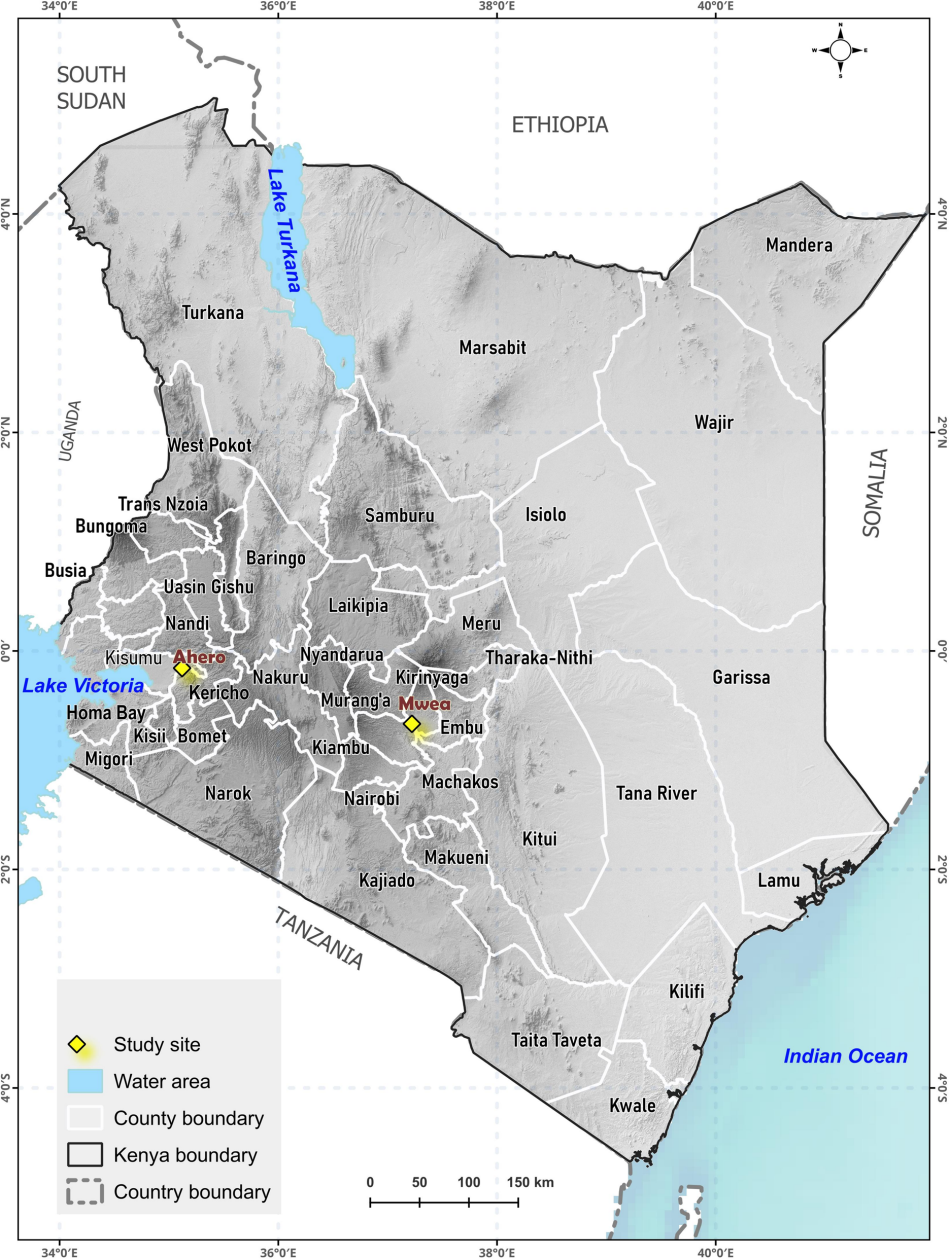
Map of Kenya showing the study sites. Geographic location of the two study areas, Ahero (Kisumu County, western Kenya) and Mwea (Kirinyaga County, central Kenya). The map was generated using Quantum Geographic Information System (QGIS) version 3.40.10. Administrative boundaries were obtained from the Global Administrative Areas (GADM) database

Mosquito samples were collected from selected villages surrounding two major rice irrigation schemes in Kenya: Mwea (0°40′S, 37°18′E) and Ahero (0°9′24″S, 35°11′54″E) (Fig. 1). The Mwea Irrigation Scheme is in Mwea Division, Kirinyaga County, approximately 100 km northeast of Nairobi, an area with seasonal malaria transmission. Detailed descriptions of the study area have been provided in previous studies [32]. The region lies at an altitude of 1000–1200 m above sea level and receives annual rainfall ranging from 356 to 1626 mm, with a mean of approximately 950 mm. The long rainy season occurs from March to May, while the short rainy season spans from October to December. The area has an average annual temperature of 21.3 °C, with minimum and maximum temperatures ranging between 16.0 and 26.5 °C, respectively. Relative humidity averages 59.5%, with 52.0% and 67.0% fluctuations. The irrigation scheme spans approximately 30,050 acres and is fed by two primary rivers, the Thiba and Nyamindi, with a combined water intake of 18.14 m^3^/s [33]. *Anopheles arabiensis* is the principal malaria vector in Mwea and is the only member of the *An. gambiae* complex that has been identified in this region [32, 34]. Recent studies in Mwea have reported resistance of *An. gambiae* (s.l) to the conventional insecticides, especially deltamethrin and alphacypermethrin [27, 29]. The most commonly used insecticides for agricultural purposes include organophosphates and a pyrethroid, alpha-cypermethrin [15]. The Ahero Rice Irrigation Scheme is located approximately 24 km east of Kisumu, along the shores of Lake Victoria in western Kenya, an area classified as malaria endemic. The scheme encompasses about 10,810 acres of irrigated land and supports over 30,000 farmers. The region experiences bimodal rainfall patterns, with long rains occurring from March to May and short rains from September to December. The region lies at an altitude of 1162–1360 m above sea level and receives an annual rainfall of between 1000 and 1800 mm. Mean annual temperatures range from 17 to 32 °C. Owing to proximity to Lake Victoria, the area is relatively humid, with an average relative humidity of approximately 65% [35]. *An. arabiensis*, and *An. funestus* are the dominant malaria vectors in Ahero [36]. Malaria control in this area has historically depended on pyrethroid-based insecticide-treated nets, and pyrethroids also remain the most used insecticides in agricultural activities, followed by organophosphates [31]. Previous reports on insecticide resistance indicate that *Anopheles gambiae* (s.l.) is resistant to several conventional insecticides [30].

### Mosquito sampling

Sampling was conducted during the 2024 long rains (May–July) in Mwea and during the short rains (October–December) in Ahero, periods that are typically associated with high mosquito densities due to increased availability of larval habitats. Blood-engorged and gravid females of *An. gambiae *sensu lato* (*s.l.*)* were collected inside human dwellings between 06:00 and 09:00 h using a Prokopack aspirator (Model 1419; John W. Hock, Gainesville, FL, USA). The collections were performed after obtaining verbal consent from household heads. In each site, three surrounding villages within the rice irrigation schemes were selected: Karima, Kariua, and Mbuinjeru in Mwea; and Nakuru, Nairobi, and Gatundu in Ahero. The villages were within the deme range of mosquitoes in *An. gambiae* s.l. In each village, at least 200 mosquitoes were collected in 10 randomly selected households. Sampled anophelines were morphologically confirmed as *An. gambiae *sensu lato (s.l.) and the mosquitoes were placed in 50 × 50 × 50 cm BugDorm cages (BugDorm, Taiwan) with ad libitum access to 10% glucose water solution and transported to the insectary at the International Centre of Insect Physiology and Ecology (ICIPE), Duduville Campus, Nairobi.

### Mosquito rearing

In the insectary, the identified blood-fed *An. gambiae* s.l. females were transferred to new 50 × 50 × 50 cm BugDorm cages (BugDorm, Taiwan). Mosquitoes collected from the three villages within each study site were pooled, and the insectary conditions were maintained at a constant temperature of 25 ± 3 °C, relative humidity of 70–80%, and a 12:12 h light–dark photoperiod. Adult females were provided ad libitum access to a 10% glucose solution. Oviposition cups lined with moist filter papers were placed centrally in each cage to facilitate egg laying. Eggs were hatched by synchronous flooding with distilled water in rearing trays (25 × 20 × 14 cm). Larvae were maintained at densities of 150–200 per tray and fed daily with fish meal flakes (Tetramin^®^; Tetra Werke, Melle, Germany). Pupae were collected using pipettes and transferred to plastic cups placed in 50 × 50 × 50 cm emergence cages for adult eclosion. The resulting first filial generation (F_1_) adults were used for subsequent insecticide susceptibility bioassays.

### Insecticide susceptibility tests

The CDC bottle bioassay kit for insecticide susceptibility tests was provided by the U.S. Department of Health and Human Services, Centers for Disease Control and Prevention (CDC), and the assays were conducted according to CDC guidelines [37]. Our preliminary assays revealed that the mosquito populations from our study sites were more sensitive to CDC bottle bioassay compared with WHO tube test. Therefore, we chose to use the CDC bottle bioassay instead of WHO tube test because we wanted to obtain samples that would later be used for transcriptomic studies to determine the mechanisms of resistance. We reasoned that using CDC bottle bioassays would limit the probability of false positives. For each insecticide tested, stock solutions were prepared by diluting technical grade insecticides in 50 mL of acetone. Each 250 mL Wheaton bottle and its corresponding cap were coated with 1 mL of the insecticide solution by rolling and inverting the bottle to ensure even distribution. The final concentrations used were: 12.5 µg/bottle for alpha-cypermethrin, deltamethrin, and bendiocarb; 21.5 µg/bottle for permethrin; 100 µg/bottle for DDT; and 50 µg/bottle for malathion. Control bottles were coated with 1 mL of acetone only. All coating procedures were performed in a fume hood, and the bottles were left to dry overnight before use.

The 3 to 5-day old *An. gambiae* s.l. females were allowed to acclimate for 1 h in paper cups before being introduced into insecticide coated test bottles. The recommended exposure duration was 30 min for pyrethroids (alphacypermethrin, deltamethrin, permethrin), organophosphates (malathion), and carbamates (bendiocarb), and 45 min for the organochloride DDT. Mosquitoes were observed for knockdown or mortality at 15 min intervals up to the diagnostic time. To generate additional data for time mortality curves, observations continued at 15 min intervals until all mosquitoes were dead or up to a maximum of 2 h. For each insecticide, a minimum of 100 female mosquitoes were tested, divided into four replicates of 25 individuals per bottle, along with a control group consisting of 20 mosquitoes. All assays were conducted between 12:00 and 15:00 h to minimize potential biases in gene expression due to circadian rhythms. Following exposure, both live and dead mosquitoes were preserved in RNAlater and stored at −80 °C for subsequent molecular analysis.

### CDC insecticide resistance intensity bioassay

To establish the intensity of permethrin resistance, serial concentrations (2 × , 5 × , and 10 ×) of permethrin were prepared and used for the CDC bottle assays. The bottles were coated in batches for each working concentration, to which mosquitoes were exposed as per the CDC guidelines [37]. Four replicates, each consisting of 25 unfed female adults aged 3–5 days were used for the bioassay, and a control group of 20 mosquitoes was exposed to acetone coated bottles only. The number of knocked-down mosquitoes was recorded every 15 min until all mosquitoes in the test bottles were either dead or reached 2 h after the start of the experiment. Mosquitoes were transferred to holding cups and provided access to 10% glucose solution. Determination of resistance intensity followed the standard WHO protocol [38].

### Synergist bioassays with PBO

The involvement of metabolic resistance mechanisms in pyrethroid resistance was determined by pre-exposing the test populations to piperonyl butoxide (PBO) at a concentration of 100 µg/bottle. PBO inhibits the specific activity of cytochrome P450 monooxygenases in insects. Four replicates of 25 unfed females aged 3–5 days were pre-exposed to PBO-coated CDC bottles for 1 h. After pre-exposure, the mosquitoes were held in paper cups for 1 h and then exposed to 12.5 µg/bottle deltamethrin, 21.5 µg/bottle permethrin, and 12.5 µg/bottle alphacypermethrin separately for another hour. A control group of 20 females was only exposed to acetone without any pre-exposure to the synergist. Mosquitoes were transferred separately to holding tubes and provided access to 10% glucose solution (wt: vol). Mortality was recorded after a 24 h recovery period.

### Molecular species identification

After oviposition, the progeny of field-collected *An. gambiae* s.l. females were used to identify species within the complex. Genomic DNA was extracted using the alcohol precipitation method described previously [39]. Species identification was performed using a species-specific polymerase chain reaction (PCR) assay [40] using the following primers: AR-3T (5′ GTGTTAAGTGTCCTTCTCCGTC-3′; specific for *An*. *arabiensis*), GA-3T (5′-GCTTACTGGTTTGGTCGGCATGT-3′; specific for *An. gambiae s.s*.), QD-3T (5′ GCATGTCCACCAACGTAAATCC-3′; specific for *An. quadriannulatus*) and IMP-UN (5′-GCTGCGAGTTGTAGAGATGCG-3′; common to all species) [41]. The 25 μL PCR reaction comprised 20–40 ng of DNA, 5X Green GoTaq Reaction Buffer (Promega), 25 mM MgCl_2_, 2 mM of each dNTP, 1U GoTaq DNA polymerase and 25 pmol/μL of primers AR-3T, GA-3T, QD-3T and IMP-UN. PCR cycling conditions were an initial denaturation step at 95 °C for 5 min; 35 cycles of 95 °C for 60 s, 53 °C for 30 s, and 72 °C for 30 s; followed by a final elongation step at 72 °C for 5 min. Amplified PCR products were separated by electrophoresis on 2% agarose gels stained with ethidium bromide and visualized under ultraviolet illumination. Band sizes were compared against a 100 base pair molecular ladder to determine species identity. DNA samples from laboratory-reared *An. arabiensis* and *An. gambiae* s.s. served as positive controls. A no-template control was included in each PCR run as a negative control. PCR amplicon sizes of 387 bp, 463 bp, and 153 bp/636 bp were used to identify *An. arabiensis*, *An. gambiae* s.s., and *An. quadriannulatus*, respectively.

### Molecular screening and quantification of *Microsporidia MB*

First filial generation (F_1_ progeny) of *An. arabiensis* mosquitoes from Ahero and Mwea, previously phenotyped for permethrin resistance were selected and screened for *Microsporidia MB* infection. Since all individuals exhibited resistance at the World Health Organization (WHO) diagnostic dose of permethrin (1 ×), higher concentrations (2 × and 5 ×) were employed to differentiate resistance intensity. Genomic DNA was extracted from subsets of live and dead mosquitoes using the alcohol precipitation method described [39]. Detection of *Microsporidia MB* was performed via quantitative PCR (qPCR) using a species-specific primer set: MB18SF (5′-CGC CGG CCG TGA AAA ATT TA-3′), and MB18SR (5′-CCT TGG ACG TGG GAG CTA TC-3′) targeting the small subunit ribosomal RNA gene as previously described [22, 42]. PCR reactions were prepared in a final volume of 10 µL, comprising 2 µL of HOT FIREPol Blend Master Mix Ready to Load (Solis BioDyne, Estonia), which includes HOT FIREPol DNA polymerase, 2 mM of each dNTP, and 7.5 mM MgCl_2_; 6 µL of nuclease-free water; 0.5 µL of each primer (5 pmol/µL); and 1 µL of genomic DNA. Amplification was performed under the following thermocycling conditions: an initial denaturation step at 95 °C for 5 min; 35 cycles of 95 °C for 30 s, 62 °C for 30 s, and 72 °C for 30 s; followed by a final extension step at 72 °C for 5 min. Each sample was run in duplicates, and infection was defined as a Ct value < 35 in both replicates. Samples with inconsistent amplification between replicates were retested, and those that failed to amplify upon repeat testing were classified as negative.

Infection intensity was further quantified by quantitative PCR (qPCR) using the same primer pair (MB18SF/MB18SR) and normalized to the *An. arabiensis* ribosomal protein S7 gene [43]. The S7 primers used were S7F: 5′-TCC TGG AGC TGG AGA TGA AC-3′ and S7R: 5′-GAC GGG TCT GTA CCT TCT GG-3′. Primer efficiency was calculated as previously described [44]. Amplification was performed on a MIC qPCR cycler (Bio Molecular Systems, Australia) under the following conditions: an initial denaturation at 95 °C for 15 min, followed by 35 cycles of 95 °C for 30 s, 62 °C for 30 s, and 72 °C for 30 s. Melt curve analysis was conducted from 65 to 95 °C to confirm specificity. Samples were considered negative if the cycle threshold (Ct) exceeded 35 or if the melt curve deviated from the positive control profile. Negative controls consisted of DNA extracted from insectary reared *An. arabiensis* at ICIPE, Nairobi. Isofemale samples derived from an MB-positive mother collected in Ahero in 2023 were used as a positive control.

### Data analysis

Insecticide efficacy against *An. arabiensis* populations was evaluated by calculating percentage knockdown and mortality rates according to the WHO standard procedures for insecticide susceptibility testing [38]. Mortality rates of 98–100% indicate susceptibility, 90–97% suggest possible resistance, and mortality below 90% confirms resistance. Resistance intensity is categorized into three (low, moderate, and high) following WHO criteria [38]. Low intensity resistance is defined as < 90% mortality at the diagnostic concentration (1 × DC) and > 98% mortality at 5 × DC. Moderate intensity resistance is indicated by < 90% mortality at 1 × DC, < 98% at 5 × DC, and > 98% at 10 × DC. High intensity resistance is defined as < 90% mortality at 1 × DC and < 98% mortality at both 5 × and 10 × DC. There was no need for Abbotts correction as no mortality was observed in the control groups. Fisher’s exact test of independence was used to compare the prevalence of *Microsporidia MB* among the mosquitoes that survived or succumbed to 2 × and 5 × permethrin discriminating dose. Data for microsporidia MB intensity was checked for normality and homogeneity of variances using Shapiro–Wilk normality test and did not meet the assumptions of normality even after transformation. Therefore, Mann–Whitney *U* test implemented in R software v 4.2.1 was used to determine whether the intensity of *Microsporidia MB* differed significantly between mosquitoes that survived or succumbed to 2 × and 5 × permethrin discriminating doses. All statistical analyses were considered significant at P < 0.05.

## Results

### Phenotypic resistance profile

**Fig. 2:**
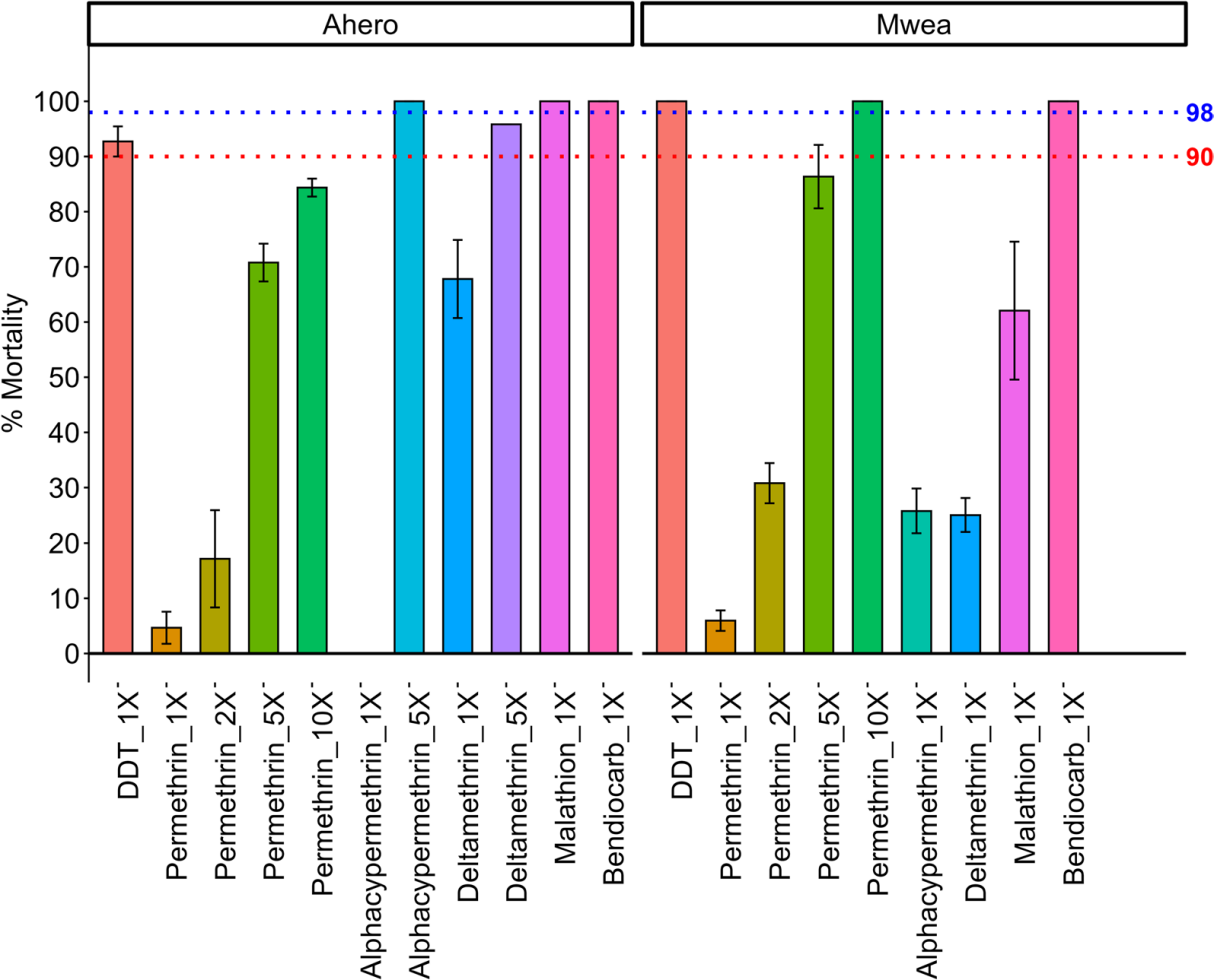
Mortality rates of *Anopheles arabiensis* population from Mwea and Ahero. Bars represent mean mortality (± SE) following exposure to diagnostic doses of organochlorine (DDT), pyrethroids (permethrin, deltamethrin, alpha-cypermethrin), an organophosphate (malathion) and a carbamate (bendiocarb). Each assay consisted of four replicates of 25 mosquitoes per insecticide per site (*n* = 100 per site per insecticide). The red dotted line marks the 90% mortality threshold for suspected resistance, while the blue dotted line indicates the 98% mortality threshold for full susceptibility, as defined by WHO criteria

A total of 3120 F_1_ females of *An. gambiae* s.l. were used for susceptibility bioassay. All the samples analyzed by PCR were identified as *An. arabiensis*. Mosquito populations from both study sites exhibited varying levels of resistance to different insecticides (Fig. 2). The mortality rates in Ahero was 0%, 4.7%, and 25.7% for alpha-cypermethrin, permethrin, and deltamethrin, respectively while corresponding mortality rates in Mwea was 6.2%, 25.7%, and 26.6% for permethrin, alpha-cypermethrin, and deltamethrin, respectively. Full susceptibility (100%) to bendiocarb was observed in *An. arabiensis* populations from both study sites. *Anopheles arabiensis* population from Ahero was susceptible to malathion, while the Mwea population exhibited resistance, with 69.2% mortality. Complete susceptibility to DDT was observed in Mwea, while suspected resistance was recorded in Ahero, with a mortality rate of 92.7%.

### Intensity of resistance

Owing to the observed resistance at the diagnostic dose (1 ×) for pyrethroids, additional bioassays were conducted using elevated concentrations (2 × , 5 × , and 10 ×) to assess the intensity of resistance in *An. arabiensis* populations. In Mwea, exposure to 5 × the diagnostic dose of permethrin resulted in a knockdown rate of 85% which increased to 100% at 10 × , indicating moderate resistance intensity (Fig. 2). In contrast, *An. arabiensis* from Ahero exhibited knockdown rates of 70.8% and 84.4% at 5 × and 10 × concentrations, respectively, suggesting a high resistance intensity (Fig. 2). In addition, moderate resistance intensity to deltamethrin and low resistance intensity to alphacypermethrin was observed in the Ahero population (Additional file 1: Supplementary Table S1).

### Synergist assays with PBO

**Fig. 3:**
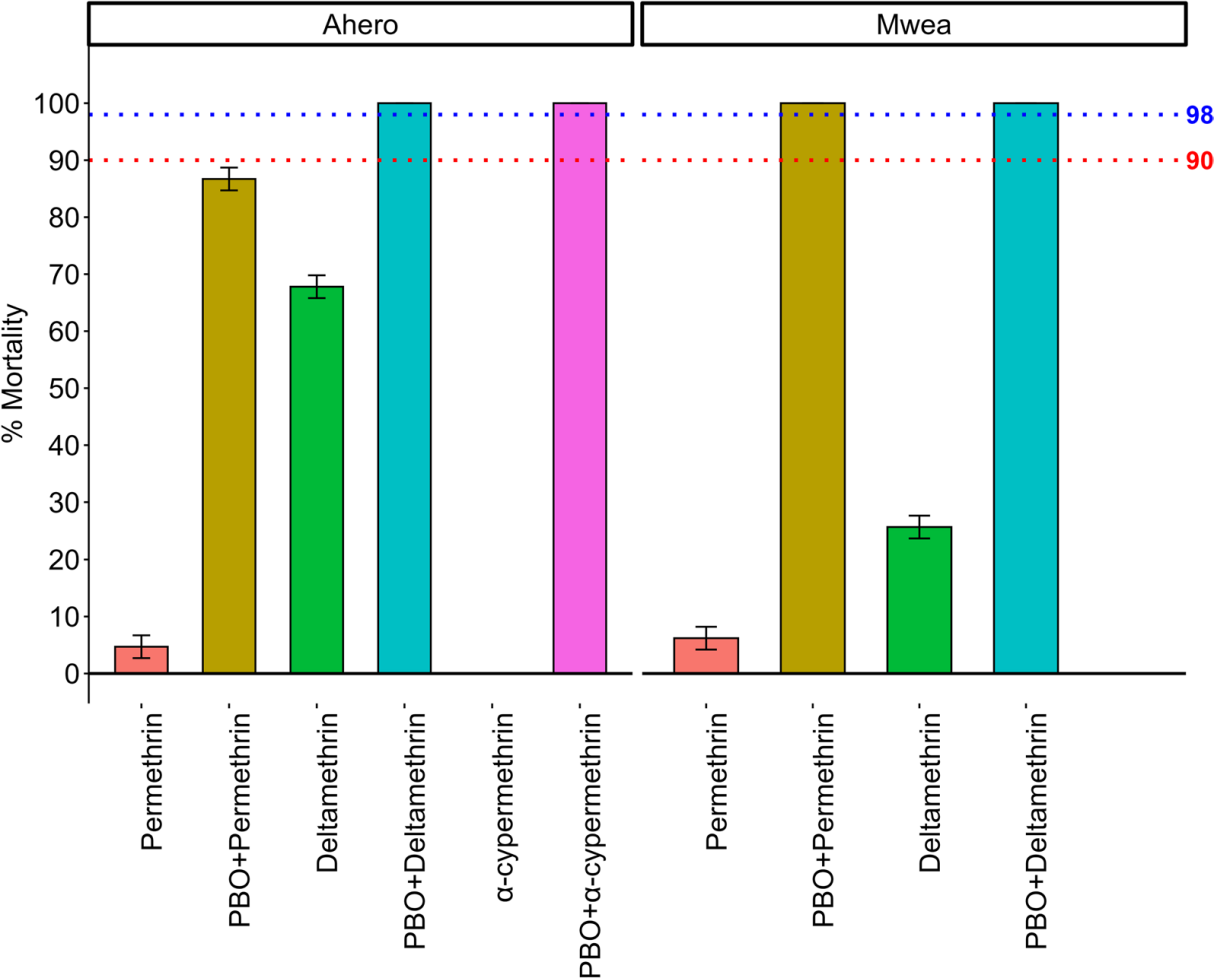
Effect of PBO synergist pre-exposure on pyrethroid susceptibility in *Anopheles arabiensis* from Ahero and Mwea. Bars show mean mortality (± SE) after pre-exposure to 4% piperonyl butoxide (PBO) followed by diagnostic doses (1 ×) of permethrin, deltamethrin, or alpha-cypermethrin. Each treatment included 100 mosquitoes per site. Each assay consisted of four replicates of 25 adult females per treatment per site (*n* = 100 mosquitoes per site per treatment). The red dotted line marks the 90% mortality threshold for suspected resistance, while the blue dotted line indicates the 98% mortality threshold for full susceptibility, as defined by WHO criteria. Synergist assays were not performed for alphacypermethrin in Mwea

The potential role of cytochrome P450s in the resistance phenotype was assessed using the synergist PBO. In Mwea, pre-exposure of *An. arabiensis* to PBO followed by deltamethrin increased mortality from 25.7% to 100%, and from 6.2% to 100% with permethrin, suggesting that cytochrome P450s are implicated in the resistance phenotype (Fig. 3). In Ahero, pre-exposure to PBO partially restored susceptibility, increasing mortality from 4.7% to 86.7% with permethrin, from 67.8% to 100% with deltamethrin, and from 0% to 100% with alphacypermethrin (Fig. 3).

### Association between *Microsporidia MB* infection and permethrin resistance intensity

**Fig. 4:**
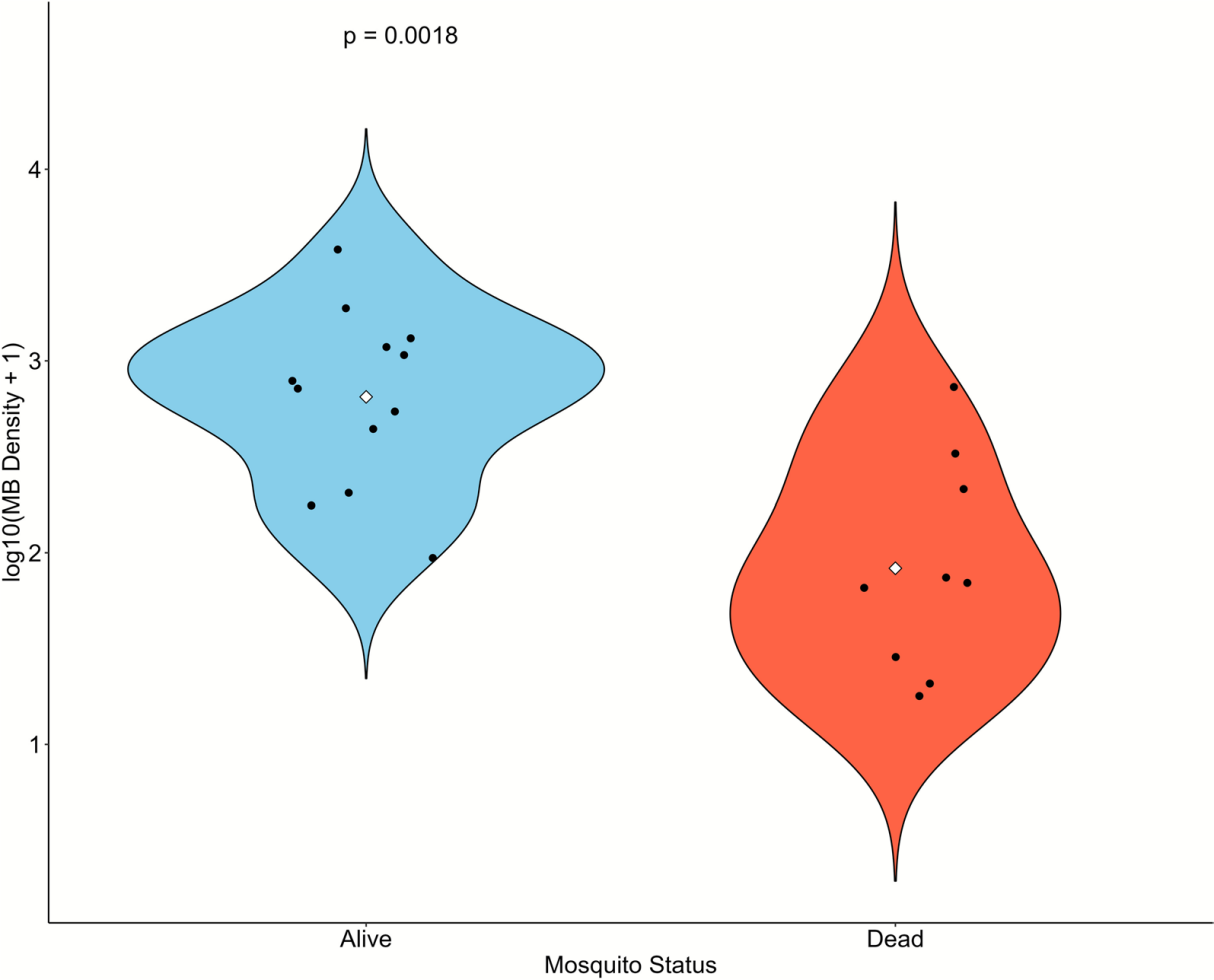
Distribution of *Microsporidia MB* density (log10-transformed) in *Anopheles arabiensis* samples that survived or succumbed to 2 × and 5 × discriminating doses of permethrin. Statistical comparisons between groups were performed using the Mann–Whitney *U* test; significance levels (*P* = 0.002) are indicated above bars. Mean values are shown as white diamonds

A subset of *An. arabiensis* samples from both study sites that survived (resistant) or succumbed (susceptible) to 2 × and 5 × discriminating doses of permethrin were screened for *Microsporidia MB* infection and density. No *Microsporidia MB* infection was detected in the Mwea population but in Ahero, the prevalence of *Microsporidia MB* infection in mosquitoes that survived exposure to 2 × and 5 × permethrin exposure was 24% (12/50) and was not significantly different from 19% (9/48) in mosquitoes that succumbed to these permethrin doses (Fisher’s exact test, *P* = 0.06, 95% CI 0.91–1.58). A total of 15 positive samples were excluded from further analysis owing to insufficient DNA concentration for qPCR. *Microsporidia MB* intensity was significantly higher in mosquitoes that survived exposure to 2 × and 5 × permethrin doses compared with those that succumbed to these doses (Mann–Whitney *U* = 96, *Z* = 2.95, *P* = 0.002) (Fig. 4).

## Discussion

This study evaluated the insecticide resistance status of *An. arabiensis* populations from two irrigated rice agroecosystems characterized by high vector densities but different malaria transmission intensities. Ahero is a malaria-endemic region characterized by perennial transmission and consistently high malaria incidence, while Mwea has seasonal transmission with relatively few malaria cases reported annually [27, 45]. *Anopheles arabiensis* was the only sibling species of *An. gambiae* s.l. identified in both study sites, consistent with reports of its ecological adaptability to irrigated habitats in East Africa [15, 46].

*Anopheles arabiensis* populations from both study sites were resistant to three pyrethroids (permethrin, deltamethrin, and alphacypermethrin) while the Ahero population showed incipient resistance to DDT, indicating emerging cross-resistance. Earlier surveys in Mwea reported full susceptibility of *An. arabiensis* to all insecticides in 2004 [28], but recent studies in this region have documented resistance of this mosquito species to pyrethroids and bendiocarb [27, 29]. These findings highlight the spatial and temporal heterogeneity of insecticide resistance in *An. arabiensis* populations.

Pyrethroids and organochlorines (e.g. DDT) act on voltage-gated sodium channels, and knockdown resistance *(kdr*) mutations in this gene can cause resistance to both insecticide classes [47]. Some cytochrome P450 enzymes (e.g., CYP6M2) can also metabolize both DDT and pyrethroids [48]. Our findings suggest the potential for cross resistance between pyrethroids and DDT in Ahero but not in Mwea population. Thus, DDT remains a viable option for IRS use in Mwea, but more studies are needed regarding its potential use in western Kenya. The observed low-to-high resistance intensity to pyrethroids aligns with resistance patterns reported in Bondo, Busia, and Kisumu [27, 49–53], confirming that pyrethroid resistance in western Kenya is intense and widespread, a pattern that is widely reported in other African countries [54–58].

The widespread use of pyrethroids both in vector control (LLINs and IRS) and in agriculture likely contributes to high intensity of pyrethroid resistance observed in this study. We did not conduct a survey of agricultural use of insecticides in our study sites, but earlier studies in Mwea revealed fenitrothion, dimethoate, and alpha cypermethrin to be the most commonly used insecticides [15]. Similar data is lacking for Ahero rice scheme, but studies from the neighboring regions that practice irrigated agriculture revealed that many households commonly used pyrethroids (62.2%) and organophosphates 6.8%) [31]. Additional studies are needed to establish and compare patterns of insecticide use in agriculture in the two study sites to allow comprehensive understanding of the relative contribution of agricultural pesticides to insecticide resistance in malaria vectors. This knowledge will inform development of location-specific insecticide resistance management, and integrated vector management (IPVM) strategies that harness intersectoral collaboration between ministries of agriculture, environment, and public health.

*Anopheles arabiensis* population from Ahero was susceptible to both bendiocarb and malathion, while population from Mwea was susceptible to bendiocarb and resistant to malathion. *Anopheles gambiae *s.l. population from western Kenya mostly display susceptibility to malathion and bendiocarb [31, 50, 51, 59], but a few instances of bendiocarb resistance have been documented in this region [59]. Variations from earlier studies [28, 29] could reflect spatial and temporal differences, changes in selection pressure, or methodological differences between the CDC bottle and WHO tube bioassays [29]. Insecticide resistance is often associated with fitness cost in insects, and this cost can vary between insecticide classes [60]. The observed incipient resistance to malathion in Mwea could result from target site mutation in *ace-1* gene or elevated detoxification enzyme activity, particularly monooxygenases and esterases [50, 61]. Previous studies have shown that CYP6P4 and glutathione S-transferase epsilon 2 (GSTE2) can metabolize several organophosphates including malathion, pirimiphos-methyl, and fenitrothion [62]. These findings highlight the ongoing challenge of insecticide resistance in Kenya, and the critical need for sustained resistance monitoring and implementation of effective resistance management strategies.

Pre-exposure to PBO fully restored the susceptibility to deltamethrin in both sites, indicating that resistance is largely mediated by cytochrome P450 monooxygenases. Thus, combining this insecticide with PBO in LLINs could be an effective vector control tool for *An. arabiensis* in the two study sites. However, partial restoration of susceptibility to permethrin in Ahero suggests the presence of additional resistance mechanisms [60, 63], highlighting the complexity of insecticide resistance. We are currently conducting genomic-level studies to identify the target-site mutations and genes potentially associated with resistance in each study site to inform locally appropriate resistance management strategies.

*Microsporidia MB* infection was only detected in *An. arabiensis* population from Ahero with infection density but not prevalence being significantly higher in mosquitoes that survived the 2 × and 5 × permethrin doses compared with those that succumbed to these doses. The lack of significant difference in *Microsporidia MB* prevalence between mosquitoes that died and survived exposure to 2 × and 5 × permethrin dose differs from recent studies in Nigeria and Niger where pyrethroid resistant *An. gambiae* s.l. population had significantly higher prevalence of *Microsporidia MB* compared with the susceptible population [64]. Our study is the first to link *Microsporidia MB* density with insecticide resistance, suggesting that *Microsporidia MB* density may be amplified in permethrin-resistant populations. *Microsporidia MB* infection was not detected in *An. arabiensis* population from Mwea, contrasting previous findings based on field-collected adult mosquitoes rather than F_1_ progenies [22]. Differences in methodology, seasonal fluctuations, vector population dynamics, or local transmission patterns could account for this discrepancy [22, 65]. Mosquito sampling in Ahero may have coincided with favorable environmental conditions, as temperature and relative humidity strongly influence *Microsporidia MB* occurrence [66, 67].

The exact mechanism(s) mediating increased density of the *Microsporidia MB* in mosquitoes expressing intense permethrin resistance is unclear, but we can offer a hypothesis. Insects have elaborate immune mechanisms that prevent endosymbionts from multiplying uncontrollably [68]. On the other hand, insecticide resistance is often associated with a fitness cost [69] and could potentially disrupt these immune mechanisms by redirecting limiting resources to expression of resistance traits, allowing the endosymbiont to proliferate. Further studies are needed to understand the interplay between insecticide resistance and mosquito immunity in relation to *Microsporidia MB* infection.

Some studies have shown that insecticide resistant vectors are more susceptible to malaria parasites compared with insecticide susceptible vectors [69]. Given the documented ability of *Microsporidia MB* to inhibit malaria parasites [22], further studies are needed to unravel whether the high *Microsporidia MB* density observed in permethrin resistant *An. arabiensis* could reduce their susceptibility to *Plasmodium* infections. In addition, our findings are based on a small sample size over a short sampling period. Future studies using larger sample sizes, multiple insecticides from different classes, and longer sampling periods could be more revealing. We also acknowledge that the laboratory nature of this study using F_1_ progeny may not necessarily reflect the situation in nature, since field mosquito populations are simultaneously exposed to multiple biotic and abiotic factors that were not considered in this study. However, our goal was to establish the association between *Microsporidia MB* and insecticide resistance, and insecticide susceptibility bioassays are typically conducted using 3–5 day nonblood fed adults which are difficult to obtain in the field.

## Conclusions

This study shows high phenotypic resistance to pyrethroids in *An. arabiensis* populations from Mwea and Ahero, each exhibiting a distinct pattern of resistance. The restoration of susceptibility with synergists supports the potential use of PBO-LLINs as an effective vector control strategy. The high density of *Microsporidia MB* observed in mosquitoes that survived exposure to 2 × and 5 × permethrin doses, compared with those that succumbed, warrants further investigation to determine whether *Microsporidia MB* influences permethrin resistance in this key malaria vector.

## Supplementary Information

Additional file 1: Table S1. Resistance intensity of *An. arabiensis* from Ahero and Mwea Irrigation schemes at diagnostic time.

## Data Availability

All data generated or analyzed during this study are included in this published article and its additional files.

## Abbreviations

AChE: Acetylcholinesterase
CDC: Centers for Disease Control and Prevention
CYP: Cytochrome P
DNA: Deoxyribonucleic acid
Dntp: Deoxynucleotide triphosphate
F: Phenylalanine
GST: Glutathione S-transferase
ICIPE: International centre of insect physiology and ecology
IPVM: Integrated pest and vector management
IRS: Indoor residual spraying
Kdr: Knockdown resistance
LLINs: Long-lasting insecticidal nets
L: Leucine
MgCl_2_: Magnesium chloride
PBO: Piperonyl butoxide
qPCR: Quantitative polymerase chain reaction
S: Serine
*s.l.*: Sensu lato
*s.s.*: Sensu stricto
WHO: World Health Organization
