# Supplementary material for: Insecticide resistance profiles of Anopheles arabiensis and relationship with Microsporidia MB infection in two rice agroecosystems in Kenya
Source: Parasit Vectors. 2026 Jan 22;19:84. doi: 10.1186/s13071-025-07212-0 (PMC12910883; doi:10.1186/s13071-025-07212-0)
Supplement: Supplementary file 1 — Additional file 1: Table S1. Resistance intensity of An. arabiensis from Ahero and Mwea Irrigation schemes at diagnostic time. [file 13071_2025_7212_MOESM1_ESM.docx]

Table S1 Mortality rate and resistance status for each insecticide tested in Ahero and Mwea *Anopheles arabiensis* populations.

| Site | Insecticide | Concentration | N (% mortality) | 95% CI | | Status |  |
| --- | --- | --- | --- | --- | --- | --- | --- |
| Ahero | Alphacypermethrin | 1× | 100(0) |  | 0 | Resistant | |
|  |  | 5× | 120(100) |  | 100-100 | Susceptible | |
|  | Bendiocarb | 1× | 80(100) |  | 100-100 | Susceptible | |
|  | DDT | 1× | 110(92.7) |  | 91.0-94.5 | Suspected resistant | |
|  | Deltamethrin | 1× | 160(67.8) |  | 62.4-73.2 | Resistant | |
|  |  | 5× | 70(95.8) |  | 93.2-98.4 | Suspected resistant | |
|  | Malathion | 1× | 90(100) |  | 100-100 | Susceptible | |
|  | Permethrin | 1× | 100(4.7) |  | 2.4-6.9 | Resistant | |
|  |  | 2× | 100(17.2) |  | 10.6-23.8 | Resistant | |
|  |  | 5× | 138(70.8) |  | 82.7-86.0 | Resistant | |
|  |  | 10× | 100(84.4) |  | 68.0-73.6 | Resistant | |
| Mwea | Alphacypermethrin | 1× | 205(25.7) |  | 20.8-30.5 | Resistant | |
|  | Bendiocarb | 1× | 131(100) |  | 100-100 | Susceptible | |
|  | DDT | 1× | 144(100) |  | 100-100 | Susceptible | |
|  | Deltamethrin | 1× | 105(26.6) |  | 17.0-36.1 | Resistant | |
|  | Malathion | 1× | 162(69.2) |  | 52.3-86.1 | Resistant | |
|  | Permethrin | 1× | 286(6.2) |  | 2.5-9.9 | Resistant | |
|  |  | 2× | 200(29.4) |  | 17.6-41.3 | Resistant | |
|  |  | 5× | 280(85.3) |  | 74.2-96.4 | Resistant | |
|  |  | 10× | 99(100) |  | 100-100 | Susceptible | |
|  | | | | | | |  |

N Number of mosquitoes; CI Confidence Intervals
